# As Far as the Eye Can See: Relationship between Psychopathic Traits and Pupil Response to Affective Stimuli

**DOI:** 10.1371/journal.pone.0167436

**Published:** 2017-01-24

**Authors:** Daniel T. Burley, Nicola S. Gray, Robert J. Snowden

**Affiliations:** 1 School of Psychology, Cardiff University, Cardiff, United Kingdom; 2 Department of Psychology, College of Human and Health Sciences, Swansea University, Swansea, United Kingdom; 3 Abertawe Bro-Morgannwg University Health Board, Swansea, United Kingdom; Universite de Bretagne Occidentale, FRANCE

## Abstract

Psychopathic individuals show a range of affective processing deficits, typically associated with the interpersonal/affective component of psychopathy. However, previous research has been inconsistent as to whether psychopathy, within both offender and community populations, is associated with deficient autonomic responses to the simple presentation of affective stimuli. Changes in pupil diameter occur in response to emotionally arousing stimuli and can be used as an objective indicator of physiological reactivity to emotion. This study used pupillometry to explore whether psychopathic traits within a community sample were associated with hypo-responsivity to the affective content of stimuli. Pupil activity was recorded for 102 adult (52 female) community participants in response to affective (both negative and positive affect) and affectively neutral stimuli, that included images of scenes, static facial expressions, dynamic facial expressions and sound-clips. Psychopathic traits were measured using the Triarchic Psychopathy Measure. Pupil diameter was larger in response to negative stimuli, but comparable pupil size was demonstrated across pleasant and neutral stimuli. A linear relationship between subjective arousal and pupil diameter was found in response to sound-clips, but was not evident in response to scenes. Contrary to predictions, psychopathy was unrelated to emotional modulation of pupil diameter across all stimuli. The findings were the same when participant gender was considered. This suggests that psychopathy within a community sample is not associated with autonomic hypo-responsivity to affective stimuli, and this effect is discussed in relation to later defensive/appetitive mobilisation deficits.

## Introduction

Psychopathy, or psychopathic personality, is a dangerous and costly disorder that has a substantial harmful effect on society. The disorder is characterised by interpersonal (e.g., callousness, manipulative, grandiose), emotional (e.g., lack of remorse and empathy, blunted emotional experience) and behavioural traits (e.g., impulsivity, irresponsibility). Psychopathy has been associated with abnormal emotional processing; research has identified that psychopathic individuals show reduced recognition of affective faces [1–5], attenuated emotional modulation of behavioural responses [6–9], deficient physiological anticipatory anxiety [10, 11], autonomic hypo-responsivity to emotion [11–15], abnormal emotional modulation of the startle response [13, 16–22], as well as abnormal brain responses to emotion [23–34]. Furthermore, these deficits exist across both negative and positive affect, although greater emotional abnormalities may occur in response to aversive cues [35, 36].

Psychopathy increasingly is being viewed as a dimensional construct rather than as a taxon [37–44] allowing researchers to investigate psychopathy within normal community samples, rather than relying on samples where the prevalence of psychopathy is high (e.g. offender or forensic psychiatric populations). Psychopathic traits in community samples have been associated with an attenuated fear-potentiated startle reflex [22, 45, 46], deficient fear conditioning [12, 47], reduced anticipatory autonomic responses [48], and reduced amygdala response to affective stimulus [15, 49–51]. Furthermore, the majority of this research has linked this hypo-responsivity to the interpersonal/affective components of psychopathy rather than the lifestyles/antisocial features [22, 45, 47–49, 52, 53], which parallels work in offender samples [13, 14, 16].

Evidence, however, is somewhat inconclusive to the nature of autonomic responses to the simple presentation of affective stimuli in psychopathy. Studies have found that psychopathy is associated with hypo-responsivity to negative stimuli only [15, 54], hypo-responsivity to all affective stimuli (both negative and positive) [55], overall smaller response magnitudes regardless of valence (including neutral images) [18, 19], or no psychopathic response deficit [16, 56]. However, it is important to delineate psychopathy into its differing dimensions given that affective deficits have been typically linked to the interpersonal/affective component of psychopathy rather than to the lifestyle/antisocial dimension.

Benning et al [45] measured skin conductance response (SCR) to moderately arousing negative and highly arousing positive images within a male community sample. The fearless dominance dimension of the Psychopathic Personality Inventory–Revised (PPI-R) [57], thought to relate to the interpersonal/affective dimension of psychopathy, was negatively associated with SCR to aversive images in comparison to neutral images, suggesting a hypo-responsivity to the affective content of the stimuli. However, the impulsive-antisocial scale, representing the lifestyle/antisocial component of psychopathy, was negatively associated with response to all images (including affectively neutral stimuli) suggesting an overall hypo-responsivity rather than a deficit specific to affective image content. Verona et al [14] presented male inmates with emotionally arousing sound-clips and found that offenders scoring high on Factor 1 (interpersonal/affective) of the Psychopathy Checklist-Revised (PCL-R) [58] had attenuated unpleasant–neutral and pleasant-neutral electrodermal differentiation, as well as reduced overall response magnitudes to all stimuli. Bate et al [59] reported that, within a male and female undergraduate sample, the interpersonal/affective dimension of psychopathy was negatively associated with SCR to negative and positive images (combined), whilst the lifestyle/antisocial factor was positively related to this emotional response, although this effect only occurred for participants lower in intelligence. Sutton et al [60] found psychopathy was related to overall SCR hypo-responsivity to all stimuli in a sample of offenders, but was unrelated to the affective content of the stimuli. Ragsdale et al (2013) [61] reported that, within a male and female community sample, both PPI-R factors and total scores were unrelated to electrodermal responses to emotionally arousing and neutral images. Therefore, while there is some evidence that psychopathy, in particular the interpersonal/affective component of the disorder, is associated with attenuated autonomic responses to the emotional content of stimuli, the presence and nature of this impairment needs further investigation.

Most studies of psychopathy have measured autonomic responses by indexing electrodermal or cardiovascular reactivity, but pupillometry offers an objective measure of emotional response that is fast, easy to administer and non-invasive (no wires are attached to the participant). The pupil is a hole located in the centre of the iris. It has a contractile structure consisting of two muscles groups controlled by opposing divisions of the autonomic nervous system: a circular group called the sphincter (or constrictor) muscle which is innervated by the parasympathetic nervous system, and the radially-arranged dilator muscle group that is activated by the sympathetic nervous system. The dynamic ‘push-pull’ between the activities of these two iris muscles determines pupil diameter. Therefore, pupil dilation can occur through both excitation of the sympathetic division or inhibition of the parasympathetic system, with the opposing activity leading to pupil constriction [62–64]. Thus, pupil diameter can act as an objective measure of autonomic arousal.

It has been proposed that affective stimuli lead to increased autonomic arousal that reflects increased defensive or appetitive motivational activation [65, 66]. Indeed, influential work by Bradley et al [63] demonstrated that the pupil is sensitive to emotion, showing greater pupil size in response to affective images of both negative and positive valence, a finding that has been replicated repeatedly [67–74]. Moreover, Bradley and colleagues [63] reported that increases in pupil diameter to emotional images is positively related to SCR, a sympathetically-mediated process [75], suggesting that affective modulation of pupil diameter indexes sympathetic excitation. Therefore, affective pupil reactivity can be viewed as an indicator of emotional processing and has been successfully applied to explore emotional processing within clinical populations [76–82], although this technique has not as yet been explored in relation to psychopathic traits.

### Current study

The present study aimed to extend previous emotion and psychopathy research by exploring whether psychopathic traits in a community sample were associated with autonomic hypo-responsivity to emotionally arousing cues using pupillometry. Psychopathy was measured using the Triarchic Psychopathy Measure (Tri-PM; [83]) a self-report measure that differentiates psychopathy into three phenotypic personality traits: Boldness, Meanness and Disinhibition. Boldness refers to the nexus of social dominance, low stress reactivity and fearlessness; Meanness reflects cruelty, lack of empathy, and excitement seeking; Disinhibition entails impulsiveness, irresponsibility, as well as impaired regulation of behaviour and affect.

The present study extends previous work by investigating pupil size to emotion across a range of stimuli types that have previously only been explored independently. First, pupil diameter was measured in response to affective images, which, as discussed earlier, has been investigated many times in normal healthy samples. However, the depiction of emotion in such scenes involves participant to process complex images which are hard to match in terms of physical properties across valences.

Second, facial expressions are thought to represent unique social and emotional stimuli [84] that are processed by a highly specialised system [85] and there is a lot of evidence that psychopathy is associated with poor processing of facial affect [86]. Facial stimuli are advantageous for pupillometry as the stimuli depicting the different valences are highly similar in terms of luminance and contrast. A number of studies have measured pupil diameter to static or morphed facial affect for adults [87–89] and children [68, 76, 81, 90]. The current study examined pupil size to facial affect by presenting images of fearful, happy, disgusted, angry and sad faces, as well as an emotionally neutral expression for comparison.

Third, while static images of faces have proved valuable affective stimuli in many studies, facial expressions are actually dynamic [91–95]. We also tested using video-clips of facial affect as they are thought to be more ecologically valid and represent the dynamic, complex, and subtle changing expressions of real-life emotion more accurately [91–95]. In addition, to our knowledge, no study has previously examined pupil response to video-clips of facial affect in adults.

Finally, we also measured pupil response to sound-clips; this is advantageous as sound is an emotionally-evocative stimulus and it removes any visual influence on the pupil, yet few studies [72, 78] have examined pupil reactivity to affective and neutral sound-clips.

Given that the pupil is thought to index emotional arousal [63], our predictions for the images and sound-clips reflected subjective arousal ratings (taken from the manuals for these stimuli). No ratings were available for the facial stimuli. We expected greater pupil diameter to the negative images and sound-clips than to pleasant or neutral stimuli. As we chose to not match the positive and negative images and sound-clips respectively on arousal, and instead chose stimuli that were unambiguously unpleasant or pleasant, this resulted in the negative valence images having greater arousal ratings than the positive images. Pleasant stimuli are hard to match to negative stimuli on arousal unless erotic cues are included [96–100], and the present study was concerned to omit erotic stimuli given future ethical concerns of showing such images to offenders. We also expected larger pupil diameter to pleasant compared to neutral images, but no difference in pupil diameter between pleasant and neutral sound-clips reflecting arousal ratings. We also predicted that the amount of dilation would be positively related to the subjective arousal-rating for the images and sound-clips. For the static and dynamic facial expressions, it was expected that negative faces would lead to larger pupil diameter than pleasant expressions as previous research has highlighted elevated autonomic responses for negative compared to happy faces suggestive of increased emotional arousal [101–104], but pleasant facial affect was still predicted to produce greater pupil size than neutral expressions.

Our main hypothesis, with regard to psychopathy, was that psychopathy would be associated with hypo-responsivity to the emotional content of the stimuli. This would be most associated with the Boldness and Meanness, but not Disinhibition, scales of the Tri-PM. This hypothesis was based on the theoretical conceptualisation of the Tri-PM dimensions of Boldness and Meanness as phenotypical expressions of fearlessness [83], alongside previous research identifying hypo-responsivity to affective stimuli being associated with interpersonal/affective traits [14, 45]. A secondary question was whether hypo-responsivity to affective stimuli was specific to negative valence, or was present for both positive and negative valences. Given the wide-range of previous findings related to this issue (see discussion above), we did not make a specific hypothesis on this matter. Finally, we also predicted that psychopathy, in particular the lifestyle/behaviour component, here represented by the Disinhibition scale, was associated with hypo-responsivity to stimuli irrespective of its affective content [14].

## Materials and Methods

### Participants

One-hundred and two participants were recruited (52 female) with a mean age of 21.08 (S.D. = 3.57) from the School of Psychology participant panel at Cardiff University from May 2014 –February 2016. Participant sample size was based on a power calculation (G*Power 3.1; [105]) for a bivariate correlation with 95% power (*α* = .05) to detect a moderate effect size consistent with previous research exploring the relationship between psychopathy and autonomic hypo-responsivity [106]. All participants had normal or corrected-to-normal vision. Participants were requested to not consume caffeine or smoke 60-minutes prior to testing. Participants were either paid money or given research credits as part of their psychology undergraduate course.

All experimental procedures were given ethical approval by the ethical committee of the School of Psychology, Cardiff University. All participants gave written informed consent to participate in the experimental procedures, and were fully debriefed at the end of the session.

### Design

Each stimuli type was presented as a separate task and every participant took part in the tasks in the same order (images, static facial expressions, dynamic facial expressions and affective sound-clips). Trials in each task began with a grey slide displaying a fixation cross (2 s) composed of alternating light and dark grey pixels whose overall luminance matched the slide luminance. The stimulus was then presented (matched for luminance to the grey slide) for a period of time (2–6 s depending on experiment) and then the grey slide (now without fixation cross) was presented until the end of the trial (total trial length 8–10 s). For the auditory sound-clip task, a blank grey screen was presented throughout the whole task with the same fixation cross displayed as described previously.

Each experiment consisted of a number of conditions that differed according to the valence of the image (for the IAPS and IADS experiments there were three conditions: unpleasant, neutral and pleasant) or the facial expression (for the static and dynamic face experiments there were 6 conditions: fear, disgust, anger, sadness, neutral, and happy).

### Measures and stimuli

#### Psychopathy measure

The Triarchic Psychopathy Measure (Tri-PM; [107]) is a 58-item self-report measure that gives participants score along the dimensions of Boldness, Meanness and Disinhibition. Meanness has been found to correlate positively with both Boldness and Disinhibition, with no relationship between Boldness and Disinhibition [108–112]. This was replicated in the present study: Meanness was positively related to Boldness, *r*(102) = .43, *p* < .001, and Disinhibition, *r*(102) = .53, *p* < .001, with no significant relationship evidenced between Boldness and Disinhibition, *r*(102) = .14, *p* = .18. The Tri-PM has shown good construct validity relating to psychopathy scores on the Psychopathy Checklist-Revised (PCL-R [58]), the most widely used psychopathy measure for assessing psychopathy within clinical populations, with Boldness and Meanness associated to Factor 1 (Affective/Interpersonal deficits) [112]. The Tri-PM is also correlated with alternative self-report psychopathy measures [113–117] including the Psychopathic Personality Inventory (PPI-R; [57]). The Tri-PM’s utility within community samples has been previously supported [116]. Moreover, the Tri-PM has been associated with good internal consistency [115, 116] and test-retest reliability [118]. The present study found that Boldness (M = 29.44, S.D. = 8.73), Meanness (M = 12.60, S.D. = 8.52) and Disinhibition (M = 14.18, S.D. = 7.50) all showed good internal reliability (Cronbach’s *α*: Boldness = .86; Meanness = .91; Disinhibition = .85).

#### Affective images

Thirty images were selected (Unpleasant: 1301, 1304, 1525, 1930, 2811, 6260, 6250, 6263, 6370, 6510; Pleasant: 1440, 1441, 1460, 1463, 1710, 1721, 1750, 2070, 4641, 8380; Neutral: 2036, 7009, 7010, 7020, 7042, 7045, 7052, 7150, 7179, 7205) from the International Affective Picture System (IAPS; [119]). We selected images that were unambiguously regarded as fear, neutral or happy based on Barke et al’s [120] affective categorisation of the IAPS images. The three conditions differed on valence using the ratings from the IAPS manual: (unpleasant = 2.94, neutral = 5.18, pleasant = 7.87; all *p*s < .001). The IAPS also provides ratings of subjective arousal for these images. The unpleasant images had the greatest arousal rating (arousal = 6.53), then the pleasant images (arousal = 4.74), with the neutral images rated least arousing neutral images (arousal = 2.90) (all *p*s < .001).

Due to the large variations in RGB colour space between the original IAPS images, all images were converted to grey-scale and equated for overall luminance using Adobe Photoshop Elements 12. Image contrast, defined as the standard deviation of all pixel values [121], was adjusted for each image and was therefore matched across valence categories. Pictures were presented on a computer display monitor and each participant was sat 57 cm from this monitor. The images subtended 50 by 30 deg. Participants were asked to simply pay attention to the images. Stimulus presentation order was randomised. Each image was presented for 2 s because we wanted a brief presentation that allowed us to assess initial emotional reaction to the stimulus while still gathering sufficient data following an expected initial stimulus-onset constriction [122].

#### Static facial expressions

Images of posed facial expression images were selected from the Radboud Faces Database [123] consisting of four male and four female actors (models chosen were 01, 02, 03, 04, 05, 07, 09 and 12). The facial expressions presented were fear, happiness, neutral, disgust, anger and sadness (48 images in total). Affective expressions showed comparable luminance and contrast values as each actor demonstrated each facial expression. There were little differences between images in RGB colour space and, therefore, facial expressions were presented in colour. All actors were presented facing forwards and with direct gaze. The images of facial affect were presented for 2 s and presentation order was randomised.

#### Dynamic facial expressions

Forty-eight video-clips were selected from the Amsterdam Dynamic Facial Expression Set (ADFES; [124]) comprised of four male and four female actors (models selected were F01, F02, F03, F05, M02, M03, M04 and M08) pulling facial expressions to fit the emotional categories of fear, happiness, neutral, disgust, anger and sadness. The videos were presented for 4 s and they depicted an actor displaying a neutral face before changing into the target expression at approximately 1.3–1.4 s post video-clip onset. Screenshots were taken from the end of each video-clip and valences showed similar luminance and contrast values). Images were similar in RGB colour space and so all video-clips were presented in colour. All actors were presented facing forwards and with direct gaze. Presentation order was randomised.

#### Affective sound-clips

We selected 30 sound-clips (Unpleasant: 106, 276, 277, 286, 291, 424, 625, 699, 711, 712; Pleasant: 110, 151, 220, 226, 230, 353, 810, 811, 815, 820; Neutral: 114, 120, 246, 320, 364, 368, 410, 425, 701, 723) from the International Affective Digitalised Sounds (IADS; [125]) consisting of 10 unpleasant sound-clips. We selected affective sound-clips that had been previously classified clearly as fearful or happy sound-clips [126], and neutral sound-clips based on their normative valence ratings: (unpleasant = 2.66, neutral = 4.90, pleasant = 7.40; all *p*s < .001). The IADS also provides ratings of subjective arousal for these images. The unpleasant images had the greatest arousal rating (arousal = 7.25) which was significantly higher (*p*s < .001), than the pleasant images (arousal = 5.52) or the neutral images (arousal = 5.52). The pleasant and neutral images did not differ significantly on arousal ratings (*p* = .33).

Sound-clips were matched across the emotional categories for maximum and average root mean square decibel level *(p*s > .05), and played to all participants at a comfortable set volume. The sound-clips were presented for 6 s and presentation order was randomised.

### Data acquisition and cleaning

A Tobii X2-60 Hz eye tracker recorded pupil data throughout each trial and allowed free movement of the head during the task. The eye trackers were calibrated to each participant’s eyes before each task using a 5-point calibration screen. The experiment took place in a dim, sound-proof room within the university.

Data was cleaned and analysed using Matlab (MathWorks, version 8.5). We removed pupil diameter increases or decreases of 0.0375 cm within a 0.02 s interval as these are thought to be artefacts [72]. We also deleted the first data point that followed missing data to avoid abnormal readings. Data for each pupil was smoothed using a low-pass Savitzky-Golay filter [127] for a span of 5 readings (over a period of approximately 0.083 s). Pupil size was determined by calculating the mean across both eyes.

### Data analysis

Trials were omitted if there was less than 50% data for the selected time window and participant means for a given emotion were only calculated if there was valid data for at least 50% of trials. Participants were excluded if they recorded less than 50% valid data across all trials during stimulus presentation. Participant’s data was identified as an outlier and removed if their data for a given valence was outside the interval defined as three times the interquartile range [128]. The sample size varied between each experiment based on the number of participants excluded with missing/outlier data (*n*: Affective images = 97; Static facial expressions = 95; Dynamic facial expressions = 92; Affective sound-clips = 97).

The baseline pupil size for each trial was calculated over the period 0.2 s prior to image-onset. For every trial, this baseline pupil diameter was subtracted from subsequent pupil size to establish baseline-corrected pupil diameter. Mean baseline-corrected pupil size was calculated over a specified time period for each task to indicate the degree of physiological responsivity.

For the affective images and static faces tasks, mean pupil size was determined from 1–2 s post-image onset. This response window period followed the initial constriction response and terminated at the end of image presentation. For the dynamic faces task we used a response window of from 3–4 s post-video-clip onset as all faces started out with a neutral expression and then began moving to the emotional expression at 1.4 s post stimulus onset (with this movement last seconds). For the affective sound-clips, no initial pupil constriction was expected as there was no visual influence on the pupil and so pupil size was calculated over the entirety of sound-clip presentation for an early (0–2 s), middle (2–4 s) and late time window (4–6 s).

Repeated measures ANOVAs were run to assess the effect of condition on pupil diameter, and planned comparison *t*-tests were conducted between each affective condition to the neutral condition. We also examined the role of participant gender by re-running the ANOVAs with gender entered as a between-subjects variable. For this gender analysis, we adopted a conservative alpha level within each task of *α* = .01 as we made no specific gender predictions.

In order to test if psychopathy was associated with reduced emotional modulation of the pupil response, we created an ‘emotional index’ for each emotion by subtracting mean neutral pupil diameter from mean pupil diameter for each affective condition. Larger values were indicative of greater emotional modulation of pupil diameter.

The relationship between Tri-PM Boldness, Meanness and Disinhibition and each emotional index was explored by conducting Pearson’s zero-order correlations and multiple linear regressions to assess the unique contribution of each dimension. Additionally, to explore the role of participant gender on psychopathy effects, we conducted hierarchical regression analyses with the dichotomous variable gender (0 = male, 1 = female) and the centred variable Tri-PM subscales entered at the first step, and the interactions between gender and each dimension entered at the second step [129]. The predicted variable was the pupil diameter emotional index across each experiment. Again, we corrected our alpha level to be more conservative (*α* = .01) within each regression as we made no specific gender predictions.

## Results

Table 1 displays mean baseline-corrected pupil diameter in response to affective and neutral stimuli for the response analysis window/s across each task. Split-half reliability checks were run finding good internal consistency for pupil size for each task with odd and even trials being highly positively related for all the visual stimuli (all *p*s < .001; Pearson’s *r*: Affective images = .81; Static facial expressions = .96; Dynamic facial expressions = .86) but were lower for the auditory stimuli (all *p*s < .001; Pearson’s *r*: Early = .40; Middle = .59; Late = .44).

**Table 1:** Mean (and standard deviations) of the pupil diameter across the response windows for the four experiments.

		Unpleasant	Pleasant	Neutral			
		*M (SD)*	*M (SD)*	*M (SD)*	*M (SD)*	*M (SD)*	*M (SD)*
Affective images		**- 0.27 (0.28)**	**- 0.39 (0.29)**	**- 0.34 (0.24)**			
		Fear	Happy	Neutral	Disgusted	Angry	Sad
Static facial expressions		**- 0.31 (0.29)**	**- 0.36 (0.29)**	**- 0.36 (0.32)**	**- 0.30 (0.30)**	**- 0.32 (0.29)**	**- 0.32 (0.29)**
Dynamic facial expressions		**0.16 (0.20)**	**0.13 (0.20)**	**0.13 (0.21)**	**0.17 (0.20)**	**0.17 (0.19)**	**0.18 (0.20)**
		Unpleasant	Pleasant	Neutral			
Affective sound-clips	Early	**0.13 (0.09)**	**0.10 (0.09)**	**0.10 (0.08)**			
	Middle	**0.28 (0.16)**	**0.17 (0.16)**	**0.17 (0.14)**			
	Late	**0.24 (0.20)**	**0.14 (0.16)**	**0.10 (0.16)**			

### Affective images

**Fig 1:**
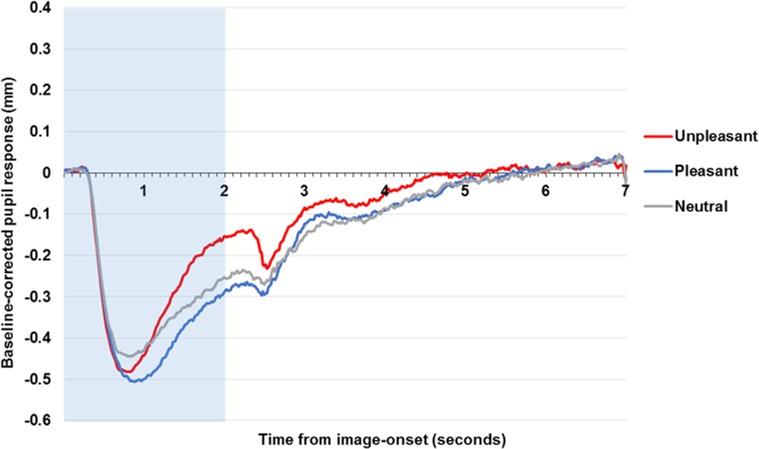
Pupil response to affective and neutral images across the community sample. Figure displays baseline-corrected pupil size to affective images. The shaded area indicates stimuli presentation.

Fig 1 shows the pupil response to the presentation of the images. There was an initial pupil constriction with an approximate latency of 0.3 s and a nadir of—0.45 mm occurring at around 0.8 s post-image onset. The pupil then increased in size until the offset of the stimulus, where there was a smaller secondary constriction, which again occurred with a latency of around 0.3 s after image-offset before recovery back to baseline pupil diameter.

It is evident that pupil diameter was modulated by image valence with unpleasant images leading to the greatest pupil size by image offset. There was a main effect of condition, *F*(2, 192) = 23.18, *p* < .001, *η*^*2*^_*p*_ = .19, with greater pupil size to unpleasant images compared to both pleasant, *t*(96) = 6.82, *p* < .001, *d* = 0.70, and neutral images *t*(96) = 3.58, *p* = .001, *d* = 0.36. Surprisingly, neutral images led to greater pupil diameter compared to pleasant images, *t*(96) = -3.21 *p* = .002, *d* = 0.33.

### Static faces

**Fig 2:**
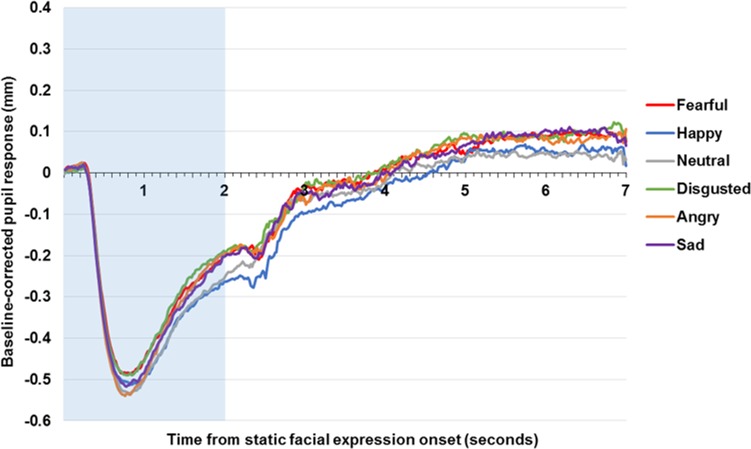
Pupil response to static affective and neutral facial expression across the community sample. Figure displays baseline-corrected pupil size to static facial expressions. The shaded area indicates stimuli presentation.

As can be seen in Fig 2, pupil response to the static facial expression parallels the pupil response pattern to the affective images. The static images led to a degree of emotional modulation, *F*(4.48, 421.32) = 4.16, *p* = .001, *η*^*2*^_*p*_ = .04. In comparison to the neutral face, the pupil was more dilated to images of faces that were fearful, *t*(94) = 2.86, *p* = .005, *d* = 0.29, disgusted, *t*(94) = 3.08, *p* = .003, *d* = 0.32, and angry, *t*(94) = 2.20, *p* = .03, *d* = 0.23, with a similar trend for sad faces *t*(94) = 1.82, *p* = .07, *d* = 0.19. However, happy and neutral faces produced no significant differences, *t*(94) = 0.02, *p* = .98, *d* = 0.002.

### Dynamic faces

**Fig 3:**
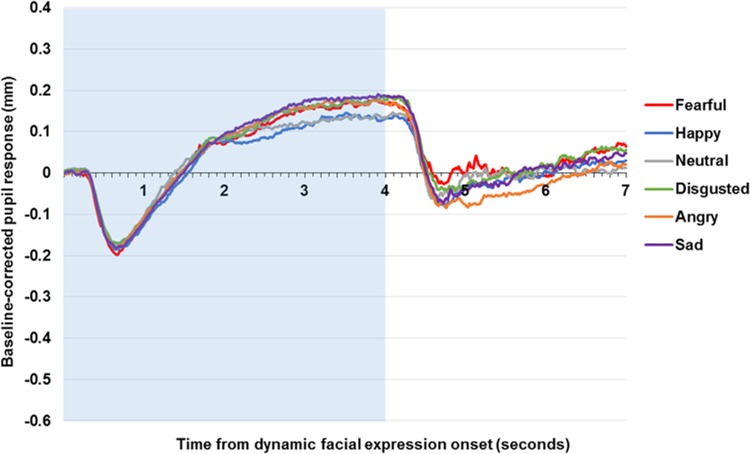
Pupil response to dynamic affective and neutral facial expression across the community sample. Figure displays baseline-corrected pupil size to dynamic facial expressions. The shaded area indicates stimuli presentation.

The dynamic facial expressions (Fig 3) led to a small initial constriction, albeit with a similar latency, reaching a nadir of approximately—0.18 mm at 0.7 s, before the pupil increased in size steadily to approximately 0.08 mm above baseline pupil diameter by 1.8 s. The increase in pupil diameter slows at 1.8 s and emotional modulation occurs at this point, which is 0.4–0.5 s after the target emotion begins on the face. Following the video-clip, the pupil constricts below baseline diameter and then appears to recover to slightly exceed baseline pupil diameter at the end of the trial, although Fig 3 does not show the full recovery period for the dynamic faces.

The dynamic faces produced a main effect of facial condition on pupil size, *F*(4.29, 390.30) = 2.51, *p* = .03, *η*^*2*^_*p*_ = .03. Compared to the neutral faces, planned comparisons showed greater pupil dilation to disgusted, *t*(91) = 2.16, *p* = .03, *d* = 0.23, angry, *t*(91) = 2.15, *p* = .03, *d* = 0.22, and sad faces, *t*(91) = 2.70, *p* = .01, *d* = 0.28, but no statistical differences to fear, *t*(91) = 1.36, *p* = .18, *d* = 0.14, or happy, *t*(91) = 0.11, *p* = .91, *d* = 0.01, faces.

### Affective sounds

**Fig 4:**
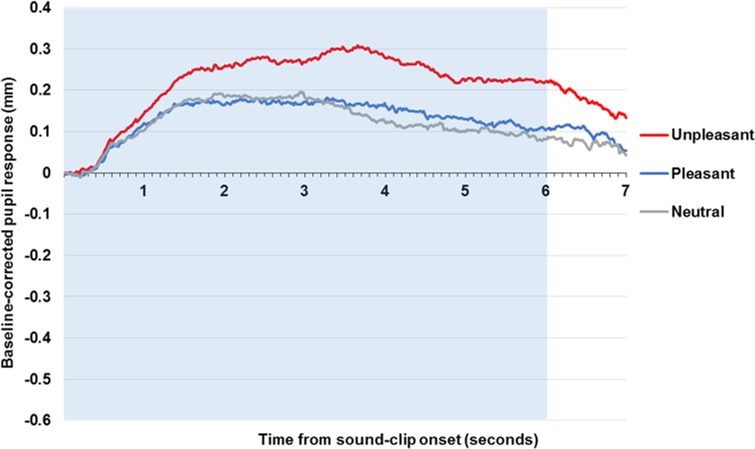
Pupil response to affective and neutral sound-clips across the community sample. Figure displays baseline-corrected pupil size to affective sound-clips. The shaded area indicates stimuli presentation.

Fig 4 demonstrates pupil reactivity across sound-clip presentation. The pupil begins to dilate with a latency of around 0.3 s, reaching a peak between 2–4 s, before pupil recovery that has not quite returned to baseline levels at 7 s, although Fig 4 does not display the full recovery period. Sound-clip valence affected pupil diameter across early, *F*(2, 192) = 8.09, *p* < .001, *η*^*2*^_*p*_ = .08, middle, *F*(2, 190) = 26.56, *p* < .001, *η*^*2*^_*p*_ = .22, and late, *F*(2, 190) = 22.47, *p* < .001, *η*^*2*^_*p*_ = .19, analysis windows. Planned comparisons identified greater pupil size in response to unpleasant sound-clips compared and neutral sound-clips (early, *t*[96] = 3.49, *p* = .001, *d* = 0.36; middle, *t*[96] = 6.12, *p* < .001, *d* = 0.62; late, *t*[96] = 5.84, *p* < .001, *d* = 0.60). No difference in pupil diameter emerged between pleasant and neutral sound-clips (early, *t*[96] = -0.25, *p* = .80, *d* = 0.03; middle, *t*[96] = 0.33, *p* = .74, *d* = 0.03; late, *t*[96] = 1.840, *p* = .07, *d* = 0.19).

### Effects of gender

Across all tasks, there was no main effect of participant gender on pupil diameter (*p*s > .08) see S1 Table) nor did any interaction surpass adjusted significance (*α* = .01).

### Subjective valence and arousal

Previous research has suggested that pupil response is driven by arousal rather than the valence of the stimuli [63]. To assess the degree to which arousal led to the observed changes in pupil diameter, we explored whether pupil diameter was related to the normative valence and/or the normative arousal ratings (scored on a 9-point scale with higher scores representing positive valence and high arousal respectively; data taken from [119, 125]). For the affective images, regression analyses for both the valence ratings and for the arousal ratings showed that neither the linear nor quadratic component was significant (*p*s > .26). For the affective sounds, valence produced a strong quadratic component (*p* = .02 for the early component, *p*s < .01 for middle and late components) while arousal produced a strong linear component (all time epochs: *p* < .001). Following this, both subjective arousal and the quadratic subjective valence term (subjective valence centred and squared) were entered into regression analyses to assess unique predictive contributions towards pupil diameter to sound-clips. Across each time-window, only subjective arousal predicted pupil diameter (*p*s < .006) with the quadratic subjective valence term not predictive (*p*s > .51).

### Effects of psychopathy

Table 2 displays the relationship between participant’s score on the Tri-PM subscales and their pupil diameter emotional indexes across each task (the difference between pupil diameter in response to affective versus neutral stimuli).

**Table 2:** Summary of zero-order correlations and multiple regression analysis between the subscales of the Triarchic Psychopathy Measure and emotional indexes for baseline-corrected pupil diameter in response to affective images, static and dynamic faces, as well as sound-clips.

			Boldness		Meanness		Disinhibition		
			*r*	*β*	*R*	*β*	*r*	*β*	*R*^*2*^
Affective images		Unpleasant	**-.02**	**-.02**	**-.03**	**.02**	**-.07**	**-.08**	**.01**
		Pleasant	**.02**	**-.01**	**.05**	**.07**	**-.001**	**-.04**	**.003**
Static facial expressions		Fearful	**.03**	**.01**	**.00**	**.13**	**-.17**	**-.24**	**.04**
		Happy	**.07**	**.02**	**.11**	**.13**	**.01**	**-.06**	**.02**
		Disgusted	**-.04**	**-.10**	**.05**	**.16**	**-.05**	**-.12**	**.02**
		Angry	**-.01**	**.01**	**-.06**	**-.05**	**-.05**	**-.02**	**.003**
		Sad	**.03**	**.03**	**-.00**	**-.001**	**-.03**	**-.03**	**.002**
Dynamic facial expressions		Fearful	**-.01**	**.02**	**-.05**	**-.03**	**-.07**	**-.06**	**.01**
		Happy	**.10**	**.10**	**.05**	**.02**	**-.01**	**-.04**	**.01**
		Disgusted	**.03**	**.05**	**-.02**	**-.04**	**-.01**	**.002**	**.002**
		Angry	**-.05**	**-.10**	**.07**	**.13**	**.03**	**-.03**	**.01**
		Sad	**.06**	**.06**	**.03**	**.003**	**.01**	**.003**	**.004**
Affective sound-clips	Early	Unpleasant	**.09**	**.09**	**.02**	**.02**	**-.05**	**-.07**	**.01**
		Pleasant	**.02**	**.08**	**-.10**	**-.16**	**-.02**	**.05**	**.02**
	Middle	Unpleasant	**.01**	**-.003**	**-.003**	**.06**	**-.09**	**-.12**	**.01**
		Pleasant	**-.02**	**.05**	**-.14**	**-.14**	**-.09**	**-.03**	**.02**
	Late	Unpleasant	**.06**	**.06**	**.02**	**.04**	**-.05**	**-.08**	**.01**
		Pleasant	**.02**	**.12**	**-.19**	**-.25**	**-.11**	**.01**	**.05**

*p < .05.

Our first hypothesis was that the emotional modulation of the pupil, here represented as the emotional index, would be weaker in those scoring highly on Boldness and Meanness specifically. Pearson’s zero-order correlations revealed that Boldness, Meanness and Disinhibition were all unrelated to emotional index to either unpleasant or pleasant images, and a multiple linear regression demonstrated that the three Tri-PM subscales failed to predict any emotional index either collectively or uniquely (this was the case without correction for multiple-tests). This lack of a significant relationship between the Tri-PM scales and the emotional index was found across all tasks (see Table 2).

Hierarchical regression analyses examined whether participant gender moderated the relationship between Tri-PM subscales and emotional index for each task. At the first step, Tri-PM total score and participant gender failed to predict the emotional indexes across any stimuli, apart from female participants showing greater modulation of pupil size to unpleasant sound-clips during the late time-window. Importantly, no interaction between participant gender and Tri-PM total score in the second step was significant. These variables did not significantly improve the predictive model across any of the four tasks, indicating that Boldness, Meanness and Disinhibition were unrelated to pupil diameter to emotional stimuli regardless of participant gender. Details are available in S2 Table.

A further hypothesis was that psychopathy, specifically Disinhibition, might be related to an overall hypo-responsivity that was not related to the affective content of the stimulus. To test this we correlated the three scales of the Tri-PM with baseline corrected pupil diameter to the neutral stimuli alone in our four experiments. No correlation approached significance (-.06 > *r*s < .12, *p*s >.24).

## Discussion

In line with previous studies [63], we found that the pupil was larger when presented with a stimulus with affective content in comparison to an emotionally neutral stimulus, although this was specific to negative stimuli. We hypothesised that the interpersonal/affective components of psychopathy would be associated with an insensitivity to the affective content of the stimuli and would therefore show less emotional modulation of the pupil. No such evidence was found. We also hypothesised that the lifestyle/antisocial aspect of psychopathy would be related to a general hypo-arousal to all stimuli (including the neutral ones). Again, we found no evidence to support this hypothesis.

### Psychopathic traits and pupil activity

We found that interpersonal/affective psychopathic traits were unrelated to emotional responses to images, static facial expressions, dynamic facial expressions and sound-clips as measured by changes in pupil diameter. This contrasts with research that found changes in emotional responses using SCRs [14, 45]. It might be typically argued that this discrepancy is a result of sample gender as both SCRs studies [14, 45] recruited male participants only, whereas the present sample consisted of both male and female participants; indeed, previous studies that have recruited female participants [60, 61] have also failed to demonstrate an association between interpersonal/affective psychopathy traits and hypo-arousal to emotional stimuli. Previous research has reported that psychopathy is more prevalent in males [43, 130] and there are reported gender differences in the conceptualisation of psychopathy [131], which may account for specific-gender effects. Yet, this explanation seems ultimately unlikely for the present data as we found no interaction between participant gender and Tri-PM scores in predicting emotional modulation of pupil diameter.

Moreover, as described in the introduction, the presence and nature of psychopathic impairments to the simple presentation of emotionally arousing stimuli has been inconsistent. These conflicting findings may reflect that psychopathic individuals are not wholly deficient in their initial autonomic response to emotion, but rather it is how they use this early information to activate appropriate defensive and appetitive motivational systems [65] that is abnormal. In support, several studies have found psychopathy to be associated with normal emotional modulation of electrodermal responses to emotion (or reduced overall SCR), but this is followed by abnormal affective potentiation of the startle response [16–19, 60], thought to reflect the underlying action disposition of the individual [132]. Therefore, it could be argued that psychopathic individuals can show normal initial autonomic response to affective cues, but rather that these cues fail to activate defensive or appetitive mobilisation.

### Emotion and pupil size across the tasks

The present data indicated that unpleasant stimuli led to greater emotional response (i.e. larger pupil dilation) than pleasant and neutral stimuli across all stimuli. This is consistent with previous psychophysiological studies showing that unpleasant affect causes elevated autonomic responses compared to pleasant affect, unless erotic stimuli are employed [96–100]. Indeed, there is evidence that the amygdala, which is central to the generation of pupil reactivity to emotion [133–136], demonstrates preferential responsivity to negative compared to positive affect [101, 137–143], and as the amygdala has been proposed as an encoder of the representation of value [144] this could suggests that pleasant affect fails to hold the same motivational value as negative affect. However, we urge caution in using the present data to support such a notion in relation to the affective images and sound-clips as we did not match the negative and positive images/sounds in terms of subjective arousal. The differences may simply reflect the greater arousal ratings of the unpleasant stimuli in comparison to the pleasant stimuli. Consistent with this, previous studies that have identified elevated pupil size to both negative and positive images [63, 78, 145] and sound-clips [72] have employed stimuli matched for subjective arousal ratings. Indeed, the present data showed that pupil diameter to the sound-clips was predicted by subjective arousal ratings uniquely over subjective valence.

However, we did not observe a linear relationship between subjective arousal and pupil diameter during the images. This may suggest that emotional arousal to images, as indexed by pupil diameter [63], and subjective arousal may not be equivalent. Weinberg and Hajcak (2010) [96] used electromyography to highlight several discrepancies between self-reported arousal and physiological responses to emotion, arguing that physiological reactivity is determined not only by perceived arousal to an affective stimulus, but also by the motivational significance of that stimulus. They found that erotic images led to brain potentials disproportionately larger than those elicited by affiliative (e.g. cuddly animals, smiling faces) and exciting images (e.g. exciting sports) despite similar subjective arousal. They suggest that exciting and affiliative images do not convey survival-relevant information and, therefore, fail to trigger motivational systems. The present study employed solely affiliative images for the pleasant category, which may explain the failure to observe an autonomic advantage to the pleasant compared to the neutral images. Furthermore, it has been found that substantial increases in SCR only occur in response to the most arousing images [100] suggesting that there is a threshold for motivation activation, which the current pleasant images may not have surpassed.

Alternatively, the failure to find a linear relationship between subjective arousal and pupil response may reflect the influence of the pupil light reflex on pupil diameter. The magnitude of the pupil light reflex primarily reflects visual stimulus factors with increased constriction to greater brightness or luminance contrast [122]. Hence, measuring pupil diameter immediately following the light reflex is likely to dilute any relationship between subjective arousal towards a stimulus and subsequent pupil diameter. Indeed, previous studies that have identified a positive relationship between subjective arousal and pupil response to images have presented images for longer durations where the influence of the light reflex on pupil diameter will be diminished [63, 146]. Further research will be needed to elucidate the precise relationship between perceived arousal and pupil response to affective images.

We also observed comparable pupil diameter between positive and neutral affect for both static and dynamic facial stimuli, despite our expectation that happy faces would induce greater emotional arousal than neutral expressions. This finding may be due to the amygdala, which has been found to be central to the generation of pupil reactivity to emotion [147], not being as responsive to happy faces as negative expressions [102]. Indeed, research indicates that happy faces are less reliant on the amygdala as patients with bilateral amygdala lesions show deficits in recognising negative faces, but no deficits in the recognition of happy faces [137, 140, 141]. The amygdala may be less central to the processing of happy faces as they do not hold motivational relevance to the individual [144] in comparison to fearful or angry faces that may signal immediate danger. An additional explanation is that neutral faces can be evaluated as negative due to an implicit social expectation for a positive facial expression [148, 149]. This is particularly pertinent in response to dynamic faces where a lack of movement conveys negative emotion [150]. It could be speculated that this perceived negativity could have contributed to physiological arousal that led to comparable pupil diameter between pleasant and neutral facial affect.

Participant gender largely showed little effect on pupil diameter to emotional and neutral stimuli across tasks. This fits with previous research that finds comparable SCR between genders in response to affective stimuli [151–153].

### Limitations and future directions

The present study used a community sample of mainly undergraduate students. Therefore the overall levels of psychopathy were low. The affective deficits of psychopaths are typically thought of as difficult to detect [154] may not manifest at lower levels of psychopathy. Several community studies that have found psychopathy is associated with autonomic hypo-responsivity to emotion [15, 45, 55, 59] have specifically recruited individuals who show high psychopathic traits to maximise the effects of psychopathy [45, 55]. This suggests that researchers may need to explore the more extreme end of the psychopathy spectrum to identify psychopathic affective deficits within normal populations. Indeed, Coid and Yang (42) reported that, within a large British community sample, psychopathy was a dimensional construct until a threshold where there was a dramatic increase in social and behavioural difficulties, and it could be argued that the same threshold might exist for affective deficits.

## Supporting Information

S1 Table Table displaying the results of mixed model ANOVAs exploring the role of gender on pupil diameter in response to affective and neutral stimuli across each task. (DOCX)

S2 Table Table displaying the results of hierarchical regressions exploring gender as a moderating variable between Tri-PM subscales and pupil diameter emotional indexes across each task. Gender was dummy coded (males = 0, females = 1).
(DOCX)

## References

[1] Blair R, Mitchell DGV, Peschardt KS, Colledge E, Leonard RA, Shine JH, et al Reduced sensitivity to others' fearful expressions in psychopathic individuals. Pers Individ Differ. 2004;37(6):1111–22.

[2] Dolan MC, Fullam RS. Face affect recognition deficits in personality-disordered offenders: Association with psychopathy. Psychol Med. 2006;36(11):1563–9. 10.1017/S0033291706008634 16893483

[3] Hastings ME, Tangney JP, Stuewig J. Psychopathy and identification of facial expressions of emotion. Pers Individ Differ. 2008;44(7):1474–83. 10.1016/j.paid.2008.01.004 PMC3086821 21547246

[4] Blair R, Colledge E, Murray L, Mitchell DGV. A selective impairment in the processing of sad and fearful expressions in children with psychopathic tendencies. J Abnorm Child Psychol. 2001;29(6):491–8. 11761283 10.1023/a:1012225108281

[5] Bowen KL, Morgan JE, Moore SC, van Goozen SHM. Young Offenders' Emotion Recognition Dysfunction Across Emotion Intensities: Explaining Variation Using Psychopathic Traits, Conduct Disorder and Offense Severity. J Psychopathol Behav Assess. 2013;36(1):60–73. 10.1007/s10862-013-9368-z PMC3935119 24610972

[6] Williamson S, Harpur TJ, Hare RD. Abnormal processing of affective words by psychopaths. Psychophysiology. 1991;28(3):260–73. 1946892 10.1111/j.1469-8986.1991.tb02192.x

[7] Mitchell DG, Richell RA, Leonard A, Blair RJR. Emotion at the expense of cognition: psychopathic individuals outperform controls on an operant response task. J Abnorm Psychol. 2006;115(3):559–66. 10.1037/0021-843X.115.3.559 16866596

[8] Blair K, Richell R, Mitchell D, Leonard A, Morton J, Blair R. They know the words, but not the music: Affective and semantic priming in individuals with psychopathy. Biological psychology. 2006;73(2):114–23. 10.1016/j.biopsycho.2005.12.006 16574302

[9] Kosson DS, Lorenz AR, Newman JP. Effects of comorbid psychopathy on criminal offending and emotion processing in male offenders with antisocial personality disorder. J Abnorm Psychol. 2006;115(4):798–806. 10.1037/0021-843X.115.4.798 17100537

[10] Ogloff JRP, Wong S. Electrodermal and cardiovascular evidence of a coping response in psychopaths. Criminal Justice and Behavior. 1990;17(2):231–45.

[11] Ishikawa SS, Raine A, Lencz T, Bihrle S, Lacasse L. Autonomic stress reactivity and executive functions in successful and unsuccessful criminal psychopaths from the community. J Abnorm Psychol. 2001;110(3):423–32. 11502085 10.1037//0021-843x.110.3.423

[12] Flor H, Birbaumer N, Hermann C, Ziegler S, Patrick CJ. Aversive Pavlovian conditioning in psychopaths: Peripheral and central correlates. Psychophysiology. 2002;39(4):505–18. 12212643 10.1017/S0048577202394046

[13] Vaidyanathan U, Hall JR, Patrick CJ, Bernat EM. Clarifying the Role of Defensive Reactivity Deficits in Psychopathy and Antisocial Personality Using Startle Reflex Methodology. J Abnorm Psychol. 2011;120(1):253–8. 10.1037/a0021224 20973594 PMC3030683

[14] Verona E, Patrick CJ, Curtin JJ, Bradley MM, Lang PJ. Psychopathy and physiological response to emotionally evocative sounds. J Abnorm Psychol. 2004;113(1):99–108. 10.1037/0021-843X.113.1.99 14992662

[15] Blair R. Responsiveness to distress cues in the child with psychopathic tendencies. Pers Individ Differ. 1999;27(1):135–45.

[16] Patrick CJ, Bradley MM, Lang PJ. Emotion in the criminal psychopath—startle reflex modulation. J Abnorm Psychol. 1993;102(1):82–92. 8436703 10.1037//0021-843x.102.1.82

[17] Levenston GK, Patrick CJ, Bradley MM, Lang PJ. The psychopath as observer: Emotion and attention in picture processing. J Abnorm Psychol. 2000;109(3):373–85. 11016107

[18] Pastor MC, Molto J, Vila J, Lang PJ. Startle reflex modulation, affective ratings and autonomic reactivity in incarcerated Spanish psychopaths. Psychophysiology. 2003;40(6):934–8. 14986846 10.1111/1469-8986.00111

[19] Herpertz SC, Werth U, Lukas G, Qunaibi M, Schuerkens A, Kunert HJ, et al Emotion in criminal offenders with psychopathy and borderline personality disorder. Arch Gen Psychiatry. 2001;58(8):737–45. 11483139 10.1001/archpsyc.58.8.737

[20] Baskin-Sommers AR, Curtin JJ, Newman JP. Specifying the attentional selection that moderates the fearlessness of psychopathic offenders. Psychol Sci. 2011;22(2):226–34. 10.1177/0956797610396227 21245494 PMC3358698

[21] Newman JP, Curtin JJ, Bertsch JD, Baskin-Sommers AR. Attention Moderates the Fearlessness of Psychopathic Offenders. Biol Psychiatry. 2010;67(1):66–70. 10.1016/j.biopsych.2009.07.035 19793581 PMC2795048

[22] Esteller À, Poy R, Moltó J. Deficient aversive-potentiated startle and the Triarchic model of psychopathy: the role of boldness. Biological psychology. 2016;117:131–40. 10.1016/j.biopsycho.2016.03.012 27033014

[23] Kiehl KA, Smith AM, Hare RD, Mendrek A, Forster BB, Brink J, et al Limbic abnormalities in affective processing by criminal psychopaths as revealed by functional magnetic resonance imaging. Biol Psychiatry. 2001;50(9):677–84. 11704074 10.1016/s0006-3223(01)01222-7

[24] Decety J, Skelly LR, Kiehl KA. Brain response to empathy-eliciting scenarios involving pain in incarcerated individuals with psychopathy. JAMA psychiatry. 2013;70(6):638–45. 10.1001/jamapsychiatry.2013.27 23615636 PMC3914759

[25] Müller JL, Sommer M, Wagner V, Lange K, Taschler H, Röder CH, et al Abnormalities in emotion processing within cortical and subcortical regions in criminal psychopaths: evidence from a functional magnetic resonance imaging study using pictures with emotional content. Biol Psychiatry. 2003;54(2):152–62. 12873805 10.1016/s0006-3223(02)01749-3

[26] Mier D, Haddad L, Diers K, Dressing H, Meyer-Lindenberg A, Kirsch P. Reduced embodied simulation in psychopathy. The World Journal of Biological Psychiatry. 2014;15(6):479–87. 10.3109/15622975.2014.902541 24802075

[27] Prehn K, Schlagenhauf F, Schulze L, Berger C, Vohs K, Fleischer M, et al Neural correlates of risk taking in violent criminal offenders characterized by emotional hypo-and hyper-reactivity. Soc Neurosci. 2013;8(2):136–47. 10.1080/17470919.2012.686923 22747189

[28] Contreras-Rodriguez O, Pujol J, Batalla I, Harrison BJ, Bosque J, Ibern-Regas I, et al Disrupted neural processing of emotional faces in psychopathy. Soc Cogn Affect Neurosci. 2014;9(4):505–12. 10.1093/scan/nst014 23386739 PMC3989133

[29] Dolan MC, Fullam RS. Psychopathy and functional magnetic resonance imaging blood oxygenation level-dependent responses to emotional faces in violent patients with schizophrenia. Biol Psychiatry. 2009;66(6):570–7. 10.1016/j.biopsych.2009.03.019 19446795

[30] Harenski CL, Kim SH, Hamann S. Neuroticism and psychopathy predict brain activation during moral and nonmoral emotion regulation. Cognitive, Affective, & Behavioral Neuroscience. 2009;9(1):1–15. 10.3758/CABN.9.1.1 19246323

[31] Harenski CL, Edwards BG, Harenski KA, Kiehl KA. Neural correlates of moral and non-moral emotion in female psychopathy. Frontiers in human neuroscience. 2014;8:741 10.3389/fnhum.2014.00741 25309400 PMC4174863

[32] Pujol J, Batalla I, Contreras-Rodríguez O, Harrison BJ, Pera V, Hernández-Ribas R, et al Breakdown in the brain network subserving moral judgment in criminal psychopathy. Soc Cogn Affect Neurosci. 2011;7(8):917–23. 10.1093/scan/nsr075 22037688 PMC3501707

[33] Meffert H, Gazzola V, den Boer JA, Bartels AA, Keysers C. Reduced spontaneous but relatively normal deliberate vicarious representations in psychopathy. Brain. 2013;136(8):2550–62. 23884812 10.1093/brain/awt190 PMC3722356

[34] Birbaumer N, Viet R, Lotze M, Erb M, Hermann C, Grodd W, et al Deficient fear conditioning in psychopathy—A functional magnetic resonance imaging study. Arch Gen Psychiatry. 2005;62(7):799–805. 10.1001/archpsyc.62.7.799 15997022

[35] Brook M, Brieman CL, Kosson DS. Emotion processing in Psychopathy Checklist—assessed psychopathy: A review of the literature. Clin Psychol Rev. 2013;33(8):979–95. 10.1016/j.cpr.2013.07.008 24013478

[36] Larson CL, Baskin-Sommers AR, Stout DM, Balderston NL, Curtin JJ, Schultz DH, et al The interplay of attention and emotion: top-down attention modulates amygdala activation in psychopathy. Cogn Affect Behav Neurosci. 2013;13(4):757–70. 10.3758/s13415-013-0172-8 23712665 PMC3806893

[37] Edens JF, Marcus DK, Lilienfeld SO, Poythress NG Jr. Psychopathic, not psychopath: taxometric evidence for the dimensional structure of psychopathy. J Abnorm Psychol. 2006;115(1):131–44. 10.1037/0021-843X.115.1.131 16492104

[38] Guay J-P, Ruscio J, Knight RA, Hare RD. A taxometric analysis of the latent structure of psychopathy: evidence for dimensionality. J Abnorm Psychol. 2007;116(4):701–16. 10.1037/0021-843X.116.4.701 18020717

[39] Marcus DK, Lilienfeld SO, Edens JF, Poythress NG. Is antisocial personality disorder continuous or categorical? A taxometric analysis. Psychol Med. 2006;36(11):1571–81. 10.1017/S0033291706008245 16836795

[40] Murrie DC, Marcus DK, Douglas KS, Lee Z, Salekin RT, Vincent G. Youth with psychopathy features are not a discrete class: A taxometric analysis. Journal of Child Psychology and Psychiatry. 2007;48(7):714–23. 10.1111/j.1469-7610.2007.01734.x 17593152

[41] Walters GD, Duncan SA, Mitchell-Perez K. The latent structure of psychopathy a taxometric investigation of the Psychopathy Checklist–Revised in a heterogeneous sample of male prison inmates. Assessment. 2007;14(3):270–8. 10.1177/1073191107299594 17690383

[42] Coid J, Yang M. The distribution of psychopathy among a household population: categorical or dimensional? Social psychiatry and psychiatric epidemiology. 2008;43(10):773–81. 10.1007/s00127-008-0363-8 18491022

[43] Coid J, Yang M, Ullrich S, Roberts A, Moran P, Bebbington P, et al Psychopathy among prisoners in England and Wales. International Journal of Law and Psychiatry. 2009;32(3):134–41. 10.1016/j.ijlp.2009.02.008 19345418

[44] Neumann CS, Hare RD. Psychopathic traits in a large community sample: links to violence, alcohol use, and intelligence. Journal of Consulting and Clinical Psychology. 2008;76(5):893–9. 10.1037/0022-006X.76.5.893 18837606

[45] Benning SD, Patrick CJ, Iacono WG. Psychopathy, startle blink modulation, and electrodermal reactivity in twin men. Psychophysiology. 2005;42(6):753–62. 10.1111/j.1469-8986.2005.00353.x 16364071 PMC2242355

[46] Justus AN, Finn PR. Startle modulation in non-incarcerated men and women with psychopathic traits. Pers Individ Differ. 2007;43(8):2057–71. 10.1016/j.paid.2007.06.020 PMC2173698 18172515

[47] López R, Poy R, Patrick CJ, Moltó J. Deficient fear conditioning and self‐reported psychopathy: The role of fearless dominance. Psychophysiology. 2013;50(2):210–8. 10.1111/j.1469-8986.2012.01493.x 23240559

[48] Wang P, Baker LA, Gao Y, Raine A, Lozano DI. Psychopathic traits and physiological responses to aversive stimuli in children aged 9–11 years. J Abnorm Child Psychol. 2012;40(5):759–69. 10.1007/s10802-011-9606-3 22228313 PMC3375395

[49] Han T, Alders GL, Greening SG, Neufeld RW, Mitchell DG. Do fearful eyes activate empathy-related brain regions in individuals with callous traits? Soc Cogn Affect Neurosci. 2012;7:958–68. 10.1093/scan/nsr068 22021652 PMC3501700

[50] Hyde LW, Byrd AL, Votruba-Drzal E, Hariri AR, Manuck SB. Amygdala Reactivity and Negative Emotionality: Divergent Correlates of Antisocial Personality and Psychopathy Traits in a Community Sample. J Abnorm Psychol. 2014;123(1):214–24. 10.1037/a0035467 24661171 PMC4008968

[51] Rilling JK, Glenn AL, Jairam MR, Pagnoni G, Goldsmith DR, Elfenbein HA, et al Neural correlates of social cooperation and non-cooperation as a function of psychopathy. Biol Psychiatry. 2007;61(11):1260–71. 10.1016/j.biopsych.2006.07.021 17046722

[52] Vanman EJ, Mejia VY, Dawson ME, Schell AM, Raine A. Modification of the startle reflex in a community sample: do one or two dimensions of psychopathy underlie emotional processing? Pers Individ Differ. 2003;35(8):2007–21.

[53] Medina AL, Kirilko E, Grose-Fifer J. Emotional processing and psychopathic traits in male college students: an event-related potential study. International Journal of Psychophysiology. 2016. 10.1016/j.ijpsycho.2016.06.004 27302151

[54] Blair R, Jones L, Clark F, Smith M. The psychopathic individual: a lack of responsiveness to distress cues? Psychophysiology. 1997;34(2):192–8. 9090269 10.1111/j.1469-8986.1997.tb02131.x

[55] Zimak EH, Suhr J, Bolinger EM. Psychophysiological and Neuropsychological Characteristics of Non-Incarcerated Adult Males with Higher Levels of Psychopathic Personality Traits. J Psychopathol Behav Assess. 2014;36(4):542–54.

[56] Pham T, Philippot P, Reme B. Subjective and autonomic responses to emotion induction in psychopaths. L ‘Encéphale. 2000;26:45–51. 10875061

[57] Lilienfeld S, Widows M. PPI-R professional manual. Lutz, FL: Psychological Assessment Resources 2005.

[58] Hare RD. The Hare Psychopathy Checklist–Revised (PCL-R) (2nd edition). Toronto: Multi-Health Systems; 2003.

[59] Bate C, Boduszek D, Dhingra K, Bale C. Psychopathy, intelligence and emotional responding in a non-forensic sample: an experimental investigation. The Journal of Forensic Psychiatry & Psychology. 2014;25(5):600–12. 26855616 10.1080/14789949.2014.943798 PMC4720052

[60] Sutton SK, Vitale JE, Newman JP. Emotion among women with psychopathy during picture perception. J Abnorm Psychol. 2002;111(4):610–9. 12428774 10.1037//0021-843x.111.4.610

[61] Ragsdale KA, Mitchell JC, Cassisi JE, Bedwell JS. Comorbidity of schizotypy and psychopathy: Skin conductance to affective pictures. Psychiatry Research. 2013;210(3):1000–7. 10.1016/j.psychres.2013.07.027 23988134

[62] White TL, Depue RA. Differential association of traits of fear and anxiety with norepinephrine- and dark-induced pupil reactivity. J Pers Soc Psychol. 1999;77(4):863–77. 10531676 10.1037//0022-3514.77.4.863

[63] Bradley MM, Miccoli L, Escrig M, Lang PJ. The pupil as a measure of emotional arousal and autonomic activation. Psychophysiology. 2008;45(4):602–7. PubMed Central PMCID: PMC3612940. 10.1111/j.1469-8986.2008.00654.x 18282202 PMC3612940

[64] Steinhauer SR, Siegle GJ, Condray R, Pless M. Sympathetic and parasympathetic innervation of pupillary dilation during sustained processing. International journal of psychophysiology: official journal of the International Organization of Psychophysiology. 2004;52(1):77–86. 15003374 10.1016/j.ijpsycho.2003.12.005

[65] Lang PJ, Bradley MM. Emotion and the motivational brain. Biological psychology. 2010;84(3):437–50. 10.1016/j.biopsycho.2009.10.007 19879918 PMC3612949

[66] Bradley MM. Natural selective attention: Orienting and emotion. Psychophysiology. 2009;46(1):1–11. 10.1111/j.1469-8986.2008.00702.x 18778317 PMC3645482

[67] Arriaga P, Adrião J, Madeira F, Cavaleiro I, Maia e Silva A, Barahona I, et al A “dry eye” for victims of violence: Effects of playing a violent video game on pupillary dilation to victims and on aggressive behavior. Psychology of Violence. 2015;5(2):199–208.

[68] Geangu E, Hauf P, Bhardwaj R, Bentz W. Infant Pupil Diameter Changes in Response to Others’ Positive and Negative Emotions. PLoS ONE. 2011;6(11). 10.1371/journal.pone.0027132 PMC3217958 22110605

[69] Henderson RR, Bradley MM, Lang PJ. Modulation of the initial light reflex during affective picture viewing. Psychophysiology. 2014;51(9):815–8. 10.1111/psyp.12236 24849784 PMC4329731

[70] Van Steenbergen H, Band GP, Hommel B. Threat but not arousal narrows attention: evidence from pupil dilation and saccade control. Cognitive and Affective Control. 2011;2(281):1–5. 10.3389/fpsyg.2011.00281 PMC3204575 22059081

[71] Bradley MM, Lang PJ. Memory, emotion, and pupil diameter: Repetition of natural scenes. Psychophysiology. 2015;52(9):1186–93. 10.1111/psyp.12442 25943211

[72] Partala T, Surakka V. Pupil size variation as an indication of affective processing. International Journal of Human-Computer Studies. 2003;59(1–2):185–98.

[73] Babiker A, Faye I, Prehn K, Malik A. Machine Learning to Differentiate Between Positive and Negative Emotions Using Pupil Diameter. Frontiers in psychology. 2015;6. 10.3389/fpsyg.2015.01921 PMC4686885 26733912

[74] Snowden RJ, O'Farrell KR, Burley D, Erichsen JT, Newton NV, Gray NS. The pupil's response to affective pictures: Role of image duration, habituation, and viewing mode. Psychophysiology. 2016;Advance online publication. 10.1111/psyp.12668 PMC5031225 27172997

[75] Wallin BG. Sympathetic nerve activity underlying electrodermal and cardiovascular reactions in man. Psychophysiology. 1981;18(4):470–6. 7267931 10.1111/j.1469-8986.1981.tb02483.x

[76] Burkhouse KL, Siegle GJ, Woody ML, Kudinova AY, Gibb BE. Pupillary reactivity to sad stimuli as a biomarker of depression risk: Evidence from a prospective study of children. J Abnorm Psychol. 2015;124(3):498–506. 10.1037/abn0000072 26147322 PMC4573844

[77] Kuchinke L, Schneider D, Kotz SA, Jacobs AM. Spontaneous but not explicit processing of positive sentences impaired in Asperger's syndrome: Pupillometric evidence. Neuropsychologia. 2011;49(3):331–8. 10.1016/j.neuropsychologia.2010.12.026 21195104

[78] Jin AB, Steding LH, Webb AK. Reduced emotional and cardiovascular reactivity to emotionally evocative stimuli in major depressive disorder. International Journal of Psychophysiology. 2015;97(1):66–74. 10.1016/j.ijpsycho.2015.04.014 25931112

[79] Siegle GJ, Steinhauer SR, Friedman ES, Thompson WS, Thase ME. Remission prognosis for cognitive therapy for recurrent depression using the pupil: utility and neural correlates. Biol Psychiatry. 2011;69(8):726–33. 10.1016/j.biopsych.2010.12.041 21447417 PMC3951934

[80] Nuske HJ, Vivanti G, Hudry K, Dissanayake C. Pupillometry reveals reduced unconscious emotional reactivity in autism. Biological psychology. 2014;101:24–35. 10.1016/j.biopsycho.2014.07.003 25017502

[81] Sepeta L, Tsuchiya N, Davies MS, Sigman M, Bookheimer SY, Dapretto M. Abnormal social reward processing in autism as indexed by pupillary responses to happy faces. J Neurodev Disord. 2012;4:9. 22958650 10.1186/1866-1955-4-17 PMC3461481

[82] Steidtmann D, Ingram RE, Siegle GJ. Pupil response to negative emotional information in individuals at risk for depression. Cogn Emot. 2010;24(3):480–96.

[83] Patrick CJ, Fowles DC, Krueger RF. Triarchic conceptualization of psychopathy: Developmental origins of disinhibition, boldness, and meanness. Development and Psychopathology. 2009;21(3):913–38. 10.1017/S0954579409000492 19583890

[84] Fridlund AJ. Human facial expression: An evolutionary view: Academic Press; 2014.

[85] Posamentier MT, Abdi H. Processing faces and facial expressions. Neuropsychol Rev. 2003;13(3):113–43. 14584908 10.1023/a:1025519712569

[86] Dawel A, O’Kearney R, McKone E, Palermo R. Not just fear and sadness: meta-analytic evidence of pervasive emotion recognition deficits for facial and vocal expressions in psychopathy. Neuroscience & Biobehavioral Reviews. 2012;36(10):2288–304. 22944264 10.1016/j.neubiorev.2012.08.006

[87] Duque A, Sanchez A, Vazquez C. Gaze-fixation and pupil dilation in the processing of emotional faces: The role of rumination. Cognition and Emotion. 2014;28(8):1347–66. 10.1080/02699931.2014.881327 24479673

[88] Prehn K, Kazzer P, Lischke A, Heinrichs M, Herpertz SC, Domes G. Effects of intranasal oxytocin on pupil dilation indicate increased salience of socioaffective stimuli. Psychophysiology. 2013;50(6):528–37. 10.1111/psyp.12042 23551070

[89] Kret ME, Roelofs K, Stekelenburg JJ, de Gelder B. Emotional signals from faces, bodies and scenes influence observers' face expressions, fixations and pupil-size. Frontiers in Human Neuroscience. 2013;7. 10.3389/fnhum.2013.00810 PMC3866922 24391567

[90] Farzin F, Rivera SM, Hessl D. Brief report: Visual processing of faces in individuals with fragile X syndrome: An eye tracking study. Journal of autism and developmental disorders. 2009;39(6):946–52. 10.1007/s10803-009-0744-1 19399604 PMC2684976

[91] Recio G, Sommer W, Schacht A. Electrophysiological correlates of perceiving and evaluating static and dynamic facial emotional expressions. Brain research. 2011;1376:66–75. 10.1016/j.brainres.2010.12.041 21172314

[92] Weyers P, Mühlberger A, Hefele C, Pauli P. Electromyographic responses to static and dynamic avatar emotional facial expressions. Psychophysiology. 2006;43(5):450–3. 10.1111/j.1469-8986.2006.00451.x 16965606

[93] Simons RF, Detenber BH, Roedema TM, Reiss JE. Emotion processing in three systems: The medium and the message. Psychophysiology. 1999;36(05):619–27. 10442030

[94] Sato W, Fujimura T, Suzuki N. Enhanced facial EMG activity in response to dynamic facial expressions. International Journal of Psychophysiology. 2008;70(1):70–4. 10.1016/j.ijpsycho.2008.06.001 18598725

[95] Rymarczyk K, Biele C, Grabowska A, Majczynski H. EMG activity in response to static and dynamic facial expressions. International Journal of Psychophysiology. 2011;79(2):330–3. 10.1016/j.ijpsycho.2010.11.001 21074582

[96] Weinberg A, Hajcak G. Beyond good and evil: the time-course of neural activity elicited by specific picture content. Emotion. 2010;10(6):767 10.1037/a0020242 21058848

[97] Sarlo M, Palomba D, Buodo G, Minghetti R, Stegagno L. Blood pressure changes highlight gender differences in emotional reactivity to arousing pictures. Biological psychology. 2005;70(3):188–96. 10.1016/j.biopsycho.2005.01.005 16242536

[98] Most SB, Smith SD, Cooter AB, Levy BN, Zald DH. The naked truth: Positive, arousing distractors impair rapid target perception. Cognition and Emotion. 2007;21(5):964–81.

[99] Bradley MM, Codispoti M, Sabatinelli D, Lang PJ. Emotion and motivation II: Sex Differences in Picture Processing. Emotion. 2001;1(3):300–19. 12934688

[100] Bradley MM, Codispoti M, Cuthbert BN, Lang PJ. Emotion and Motivation I: Defensive and Appetitive Reactions in Picture Processing. Emotion. 2001;1(3):276–98. 12934687

[101] Breiter HC, Etcoff NL, Whalen PJ, Kennedy WA, Rauch SL, Buckner RL, et al Response and habituation of the human amygdala during visual processing of facial expression. Neuron. 1996;17(5):875–87. 8938120 10.1016/s0896-6273(00)80219-6

[102] Morris J, Frith C, Perrett D, Rowland D, Young A, Calder A, et al A differential neural response in the human amygdala to fearful and happy facial expressions. Nature. 1996;383:31. 10.1038/383812a0 8893004

[103] Leppänen JM, Kauppinen P, Peltola MJ, Hietanen JK. Differential electrocortical responses to increasing intensities of fearful and happy emotional expressions. Brain Research. 2007;1166:103–9. 10.1016/j.brainres.2007.06.060 17662698

[104] Levenson RW. Autonomic nervous system differences among emotions. Psychol Sci. 1992;3(1):23–7.

[105] Faul F, Erdfelder E, Lang A-G, Buchner A. G* Power 3: A flexible statistical power analysis program for the social, behavioral, and biomedical sciences. Behavior research methods. 2007;39(2):175–91. 17695343 10.3758/bf03193146

[106] Lorber MF. Psychophysiology of aggression, psychopathy, and conduct problems: A meta-analysis. Psychol Bull. 2004;130(4):531–52. 10.1037/0033-2909.130.4.531 15250812

[107] Patrick C. Triarchic psychopathy measure (TriPM). PhenX Toolkit Online assessment catalog. 2010.

[108] Craig RL, Gray NS, Snowden RJ. Recalled parental bonding, current attachment, and the triarchic conceptualisation of psychopathy. Pers Individ Differ. 2013;55(4):345–50.

[109] Poy R, Segarra P, Esteller A, Lopez R, Molto J. FFM Description of the Triarchic Conceptualization of Psychopathy in Men and Women. Psychol Assess. 2014;26(1):69–76. 10.1037/a0034642 24099318

[110] Strickland CM, Drislane LE, Lucy M, Krueger RF, Patrick CJ. Characterizing Psychopathy Using DSM-5 Personality Traits. Assessment. 2013;20(3):327–38. 10.1177/1073191113486691 23620353

[111] Patrick CJ, Drislane LE. Triarchic model of psychopathy: Origins, operationalizations, and observed linkages with personality and general psychopathology. J Pers. 2015;83(6):627–43. 10.1111/jopy.12119 25109906

[112] Venables N, Hall J, Patrick C. Differentiating psychopathy from antisocial personality disorder: a triarchic model perspective. Psychol Med. 2014;44(05):1005–13. 23834781 10.1017/S003329171300161X

[113] Drislane LE, Patrick CJ, Arsal G. Clarifying the content coverage of differing psychopathy inventories through reference to the Triarchic Psychopathy Measure. Psychol Assess. 2014;26(2):350 10.1037/a0035152 24320762 PMC4100942

[114] Marion BE, Sellbom M, Salekin RT, Toomey JA, Kucharski LT, Duncan S. An examination of the association between psychopathy and dissimulation using the MMPI-2-RF validity scales. Law and human behavior. 2013;37(4):219 10.1037/lhb0000008 22799603

[115] Stanley JH, Wygant DB, Sellbom M. Elaborating on the construct validity of the triarchic psychopathy measure in a criminal offender sample. Journal of Personality Assessment. 2013;95(4):343–50. 10.1080/00223891.2012.735302 23113864

[116] Sellbom M, Phillips TR. An Examination of the Triarchic Conceptualization of Psychopathy in Incarcerated and Nonincarcerated Samples. J Abnorm Psychol. 2013;122(1):208–14. 10.1037/a0029306 22867118

[117] Lilienfeld SO, Smith SF, Sauvigné KC, Patrick CJ, Drislane LE, Latzman RD, et al. Is Boldness Relevant to Psychopathic Personality? Meta-Analytic Relations With Non-Psychopathy Checklist-Based Measures of Psychopathy. 2015. 10.1037/pas0000244 26619088

[118] Blagov PS, Patrick CJ, Oost KM, Goodman JA, Pugh AT. Triarchic psychopathy measure: Validity in relation to normal-range traits, personality pathology, and psychological adjustment. J Pers Disord. 2015;29:182. 10.1521/pedi_2015_29_182 26828107

[119] Lang PJ, Bradley MM, Cuthbert B. International affective picture system (IAPS): Affective ratings of pictures and instruction manual. Technical Report A-8. University of Florida, Gainesville, FL.2008.

[120] Barke A, Stahl J, Kroner-Herwig B. Identifying a subset of fear-evoking pictures from the IAPS on the basis of dimensional and categorical ratings for a German sample. Journal of behavior therapy and experimental psychiatry. 2012;43:565–72. 10.1016/j.jbtep.2011.07.006 21839700

[121] Moulden B, Kingdom F, Gatley LF. The standard deviation of luminance as a metric for contrast in random-dot images. Perception. 1990;19(1):79–101. 2336338 10.1068/p190079

[122] Barbur J. Learning from the pupil—studies of basic mechanisms and clincal applications. The Visual Neurosciences. 2004;1:641–56.

[123] Langner O, Dotsch R, Bijlstra G, Wigboldus DHJ, Hawk ST, van Knippenberg A. Presentation and validation of the Radboud Faces Database. Cogn Emot. 2010;24(8):1377–88.

[124] van der Schalk J, Hawk ST, Fischer AH, Doosje B. Moving Faces, Looking Places: Validation of the Amsterdam Dynamic Facial Expression Set (ADFES). Emotion. 2011;11(4):907–20. 10.1037/a0023853 21859206

[125] Bradley MM, Lang PJ. The International Affective Digitized Sounds (2nd Edition; IADS-2): Affective ratings of sounds and instruction manual. Technical report B-3. University of Florida, Gainesville, Fl.2007.

[126] Stevenson RA, James TW. Affective auditory stimuli: Characterization of the International Affective Digitized Sounds (IADS) by discrete emotional categories. Behavior Research Methods. 2008;40(1):315–21. 18411555 10.3758/brm.40.1.315

[127] Savitzky A, Golay MJ. Smoothing and differentiation of data by simplified least squares procedures. Analytical chemistry. 1964;36(8):1627–39.

[128] Tukey JW. Exploratory data analysis. 1977.

[129] Aguinis H. Regression analysis for categorical moderators: Guilford Press; 2004.

[130] Coid J, Yang M, Ullrich S, Roberts A, Hare RD. Prevalence and correlates of psychopathic traits in the household population of Great Britain. International Journal of Law and Psychiatry. 2009;32(2):65–73. 10.1016/j.ijlp.2009.01.002 19243821

[131] Forouzan E, Cooke DJ. Figuring out la femme fatale: Conceptual and assessment issues concerning psychopathy in females. Behav Sci Law. 2005;23(6):765–78. 10.1002/bsl.669 16333807

[132] Lang PJ, Bradley MM, Cuthbert BN. Emotion, attention, and the startle reflex. Psychol Rev. 1990;97(3):377–95. 2200076

[133] Applegate CD, Kapp BS, Underwood MD, McNall CL. Autonomic and somatomotor effects of amygdala central N. stimulation in awake rabbits. Physiol Behav. 1983;31(3):353–60. 6635005 10.1016/0031-9384(83)90201-9

[134] Ursin H, Kaada BR. Functional localization within the amygdaloid complex in the cat. Electroencephalography and Clinical Neurophysiology. 1960;12(1):1–20. 13840488 10.1016/0013-4694(60)90058-4

[135] Gloor P. The temporal lobe and limbic system: Oxford University Press, USA; 1997.

[136] Siegle GJ, Thompson W, Carter CS, Steinhauer SR, Thase ME. Increased Amygdala and Decreased Dorsolateral Prefrontal BOLD Responses in Unipolar Depression: Related and Independent Features. Biol Psychiatry. 2007;61(2):198–209. 10.1016/j.biopsych.2006.05.048 17027931

[137] Adolphs R, Tranel D, Damasio H, Damasio A. Impaired recognition of emotion in facial expressions following bilateral damage to the human amygdala. Nature. 1994;372(6507):669–72. 10.1038/372669a0 7990957

[138] Adolphs R, Tranel D, Damasio H, Damasio AR. Fear and the human amygdala. The Journal of Neuroscience. 1995;15(9):5879–91. 7666173 10.1523/JNEUROSCI.15-09-05879.1995 PMC6577662

[139] Hamann SB, Stefanacci L, Squire LR, Adolphs R, Tranel D, Damasio H, et al Recognizing facial emotion. Nature. 1996;379(6565): 497 10.1038/379497a0 8596627

[140] Calder AJ. Facial emotion recognition after bilateral amygdala damage: differentially severe impairment of fear. Cognitive Neuropsychology. 1996;13(5):699–745.

[141] Adolphs R, Tranel D, Hamann S, Young AW, Calder AJ, Phelps EA, et al Recognition of facial emotion in nine individuals with bilateral amygdala damage. Neuropsychologia. 1999;37(10):1111–7. 10509833 10.1016/s0028-3932(99)00039-1

[142] Canli T, Sivers H, Whitfield SL, Gotlib IH, Gabrieli JD. Amygdala response to happy faces as a function of extraversion. Science. 2002;296(5576):2191 10.1126/science.1068749 12077407

[143] Somerville LH, Kim H, Johnstone T, Alexander AL, Whalen PJ. Human amygdala responses during presentation of happy and neutral faces: correlations with state anxiety. Biol Psychiatry. 2004;55(9):897–903. 10.1016/j.biopsych.2004.01.007 15110733

[144] Morrison SE, Salzman CD. Re-valuing the amygdala. Current Opinion in Neurobiology. 2010;20(2):221–30. 10.1016/j.conb.2010.02.007 20299204 PMC2862774

[145] Dietz J, Bradley MM, Okun M, Bowers D. Emotion and ocular responses in Parkinson's disease. Neuropsychologia. 2011;49(12):3247–53. 10.1016/j.neuropsychologia.2011.07.029 21839756 PMC3384545

[146] Lemaire M, Aguillon-Hernandez N, Bonnet-Brilhault F, Martineau J, El-Hage W. Subjective and physiological emotional response in euthymic bipolar patients: A pilot study. Psychiatry research. 2014;220(1):294–301. 25064388 10.1016/j.psychres.2014.07.002

[147] Siegle GJ, Steinhauer SR, Stenger VA, Konecky R, Carter CS. Use of concurrent pupil dilation assessment to inform interpretation and analysis of fMRI data. NeuroImage. 2003;20(1):114–24. 14527574 10.1016/s1053-8119(03)00298-2

[148] Thomas KM, Drevets WC, Whalen PJ, Eccard CH, Dahl RE, Ryan ND, et al Amygdala response to facial expressions in children and adults. Biol Psychiatry. 2001;49(4):309–16. 11239901 10.1016/s0006-3223(00)01066-0

[149] Lee B-T, Seok J-H, Lee B-C, Cho SW, Yoon B-J, Lee K-U, et al Neural correlates of affective processing in response to sad and angry facial stimuli in patients with major depressive disorder. Progress in Neuro-Psychopharmacology and Biological Psychiatry. 2008;32(3):778–85. 10.1016/j.pnpbp.2007.12.009 18207298

[150] Wallbott HG, Ricci‐Bitti P. Decoders' processing of emotional facial expression—a top‐down or bottom‐up mechanism? European Journal of Social Psychology. 1993;23(4):427–43.

[151] Codispoti M, Surcinelli P, Baldaro B. Watching emotional movies: Affective reactions and gender differences. International Journal of Psychophysiology. 2008;69(2):90–5. 10.1016/j.ijpsycho.2008.03.004 18433903

[152] Wrase J, Klein S, Gruesser SM, Hermann D, Flor H, Mann K, et al Gender differences in the processing of standardized emotional visual stimuli in humans: a functional magnetic resonance imaging study. Neuroscience letters. 2003;348(1):41–5. 12893421 10.1016/s0304-3940(03)00565-2

[153] Bianchin M, Angrilli A. Gender differences in emotional responses: A psychophysiological study. Physiol Behav. 2012;105(4):925–32. 10.1016/j.physbeh.2011.10.031 22108508

[154] Wilson K, Juodis M, Porter S. Fear and loathing in psychopaths: A meta-analytic investigation of the facial affect recognition deficit. Criminal Justice and Behavior. 2011;38(7):659–68.

