# Supplementary material for: As Far as the Eye Can See: Relationship between Psychopathic Traits and Pupil Response to Affective Stimuli
Source: PLoS One. 2017 Jan 24;12(1):e0167436. doi: 10.1371/journal.pone.0167436 (PMC5261620; doi:10.1371/journal.pone.0167436)
Supplement: S1 Table — (DOCX) [file pone.0167436.s001.docx]

|  | |  | ***F*** | ***η^2^_p_*** |
| --- | --- | --- | --- | --- |
| **Affective images** | | **Gender** | 3.08 | .03 |
|  |  | **Gender x Valence** | 3.18 | .03 |
| **Static facial expressions** | | **Gender** | 2.40 | .03 |
|  |  | **Gender x Valence** | 0.44 | .01 |
| **Dynamic facial expressions** | | **Gender** | 0.65 | .01 |
|  |  | **Gender x Valence** | 1.12 | .01 |
| **Affective sound-clips** | **Early** | **Gender** | 0.50 | .01 |
|  |  | **Gender x Valence** | 0.81 | .002 |
|  | **Middle** | **Gender** | 1.37 | .01 |
|  |  | **Gender x Valence** | 1.00 | .01 |
|  | **Late** | **Gender** | 0.94 | .01 |
|  |  | **Gender x Valence** | 3.77 | .04 |

*** *p* < .01, Adjusted *α* level,**
