# Supplementary material for: As Far as the Eye Can See: Relationship between Psychopathic Traits and Pupil Response to Affective Stimuli
Source: PLoS One. 2017 Jan 24;12(1):e0167436. doi: 10.1371/journal.pone.0167436 (PMC5261620; doi:10.1371/journal.pone.0167436)
Supplement: S2 Table — Gender was dummy coded (males = 0, females = 1). (DOCX) [file pone.0167436.s002.docx]

|  | | |  | **Gender** | **Boldness** | **Meanness** | **Disinhibition** | **Boldness x gender** | **Meanness x gender** | **Disinhibition x gender** |  |  |
| --- | --- | --- | --- | --- | --- | --- | --- | --- | --- | --- | --- | --- |
|  |  |  |  | ***β*** | ***β*** | ***β*** | ***β*** | ***β*** | ***β*** | ***β*** | ***R^2^*** | ***∆R^2^*** |
| **Affective images** | | **Unpleasant** | **Step 1** | .21 | .03 | .09 | -.08 |  |  |  |  |  |
|  |  |  | **Step 2** | .16 | .004 | .28 | -.17 | .09 | -.36 | .17 | .09 | .05 |
|  |  | **Pleasant** | **Step 1** | -.07 | -.02 | .05 | -.04 |  |  |  |  |  |
|  |  |  | **Step 2** | -.10 | -.05 | .20 | -.17 | .09 | -.28 | .21 | .04 | .03 |
| **Static facial expressions** | | **Fearful** | **Step 1** | .03 | .02 | .14 | -.24 |  |  |  |  |  |
|  |  |  | **Step 2** | .05 | -.01 | .08 | -.24 | .02 | .12 | -.02 | .05 | .04 |
|  |  | **Happy** | **Step 1** | .12 | .05 | .17 | -.07 |  |  |  |  |  |
|  |  |  | **Step 2** | .15 | .07 | .09 | -.07 | -.04 | .19 | -.02 | .04 | .03 |
|  |  | **Disgusted** | **Step 1** | .09 | -.07 | .19 | -.13 |  |  |  |  |  |
|  |  |  | **Step 2** | .09 | -.07 | .22 | -.22 | .01 | -.05 | .14 | .03 | .05 |
|  |  | **Angry** | **Step 1** | .15 | .05 | .004 | -.02 |  |  |  |  |  |
|  |  |  | **Step 2** | .16 | -.05 | -.05 | .01 | .11 | .12 | -.05 | .04 | .01 |
|  |  | **Sad** | **Step 1** | .09 | .06 | .03 | -.03 |  |  |  |  |  |
|  |  |  | **Step 2** | .08 | .04 | .05 | -.03 | .02 | -.04 | .001 | .01 | .02 |
| **Dynamic facial expressions** | | **Fearful** | **Step 1** | .12 | .05 | .01 | -.05 |  |  |  |  |  |
|  |  |  | **Step 2** | .12 | -.03 | .02 | -.08 | .11 | -.02 | .06 | .02 | .01 |
|  |  | **Happy** | **Step 1** | .08 | .12 | .04 | -.03 |  |  |  |  |  |
|  |  |  | **Step 2** | .11 | .23 | -.16 | -.003 | -.22 | .42 | -.15 | .08 | .07 |
|  |  | **Disgusted** | **Step 1** | .31 | .13 | .05 | .02 |  |  |  |  |  |
|  |  |  | **Step 2** | .30 | .10 | .12 | -.01 | .06 | -.14 | .07 | .08 | .01 |
|  |  | **Angry** | **Step 1** | .24 | -.04 | .20 | -.01 |  |  |  |  |  |
|  |  |  | **Step 2** | .24 | -.13 | .15 | .09 | .08 | .10 | -.15 | .07 | .01 |
|  |  | **Sad** | **Step 1** | .16 | .10 | .05 | .01 |  |  |  |  |  |
|  |  |  | **Step 2** | .17 | .08 | .04 | -.04 | .04 | .02 | .08 | .03 | .01 |
| **Affective sound-clips** | **Early** | **Unpleasant** | **Step 1** | .003 | .09 | .02 | -.07 |  |  |  |  |  |
|  |  |  | **Step 2** | .01 | .02 | .07 | -.18 | .12 | -.06 | .17 | -.05 | .02 |
|  |  | **Pleasant** | **Step 1** | .01 | .07 | -.15 | .03 |  |  |  |  |  |
|  |  |  | **Step 2** | .01 | .17 | -.21 | .16 | .14 | .08 | -.18 | .04 | .02 |
|  | **Middle** | **Unpleasant** | **Step 1** | .13 | .03 | .10 | -.12 |  |  |  |  |  |
|  |  |  | **Step 2** | .11 | -.05 | .18 | -.15 | .13 | -.15 | .08 | .03 | .01 |
|  |  | **Pleasant** | **Step 1** | .13 | .08 | -.10 | -.03 |  |  |  |  |  |
|  |  |  | **Step 2** | .11 | .13 | -.08 | .06 | -.08 | -.06 | -.13 | .06 | .02 |
|  | **Late** | **Unpleasant** | **Step 1** | .20 | .11 | .10 | -.08 |  |  |  |  |  |
|  |  |  | **Step 2** | .20 | -.03 | .13 | -.06 | .20 | -.20 | -.06 | .05 | .02 |
|  |  | **Pleasant** | **Step 1** | .32**^*^** | .20 | -.14 | -.002 |  |  |  |  |  |
|  |  |  | **Step 2** | .29 | .15 | -.12 | .15 | .06 | -.08 | -.20 | .16**^*^** | .04 |

*** *p* < .01, Adjusted *α* level,**

***β,* standardised beta,**

***R^2^* ,variance,**

***∆R^2^*, change in variance**
